# Selenium Forms and Dosages Determined Their Biological Actions in Mouse Models of Parkinson’s Disease

**DOI:** 10.3390/nu15010011

**Published:** 2022-12-20

**Authors:** Chongchong Sun, Zhongrui Du, Xin Liu, Ye Yang, Sainan Zhou, Chong Li, Xu Cao, Qing Zhao, Kahing Wong, Wenfang Chen, Xiaoli Dong

**Affiliations:** 1 Department of Physiology, School of Basic Medicine, Qingdao University, Qingdao 266071, China; 2 The Hong Kong Polytechnic University Shenzhen Research Institute, Shenzhen 518057, China; 3 Research Institute for Future Food, The Hong Kong Polytechnic University, Hong Kong 852, China; 4 Department of Applied Biology and Chemical Technology, The Hong Kong Polytechnic University, Hong Kong 852, China; 5 BioNanotechnology Institute, Ludong University, Yantai 264025, China; 6 Department of Neurology, Shenzhen University General Hospital, Shenzhen University Clinical Medical Academy, Shenzhen 518055, China; 7 Department of Neurology, Linzi Maternal & Child Health Hospital of Zibo, Zibo 255400, China

**Keywords:** Parkinson’s disease, selenium, sodium selenite, seleno-L-methionine, selenoproteins

## Abstract

Selenium (Se), an essential antioxidant trace element, is reported to play a role in Parkinson’s disease (PD). However, there is a lack of systematic studies on different Se forms against PD. Our study is designed to compare the neuroprotective effects of inorganic and organic Se in two classical PD mice models and investigate the underlying mechanisms for their potentially differential actions against PD. In this study, different dosages of inorganic sodium selenite (Se-Na) or organic seleno-L-methionine (Se-Met) were fed to either acute or chronic PD mice models, and their neuroprotective effects and mechanisms were explored and compared. Se-Na provided better neuroprotective effects in PD mice than Se-Met administered at the same but at a relatively low Se dosage. Se-Na treatment could influence GPX activities but not their mRNA expressions in the midbrains of PD mice. The enhanced GPX activities caused by Se-Na, but not Se-Met, in PD mice could be the major reason for the positive actions of inorganic Se to prevent dopaminergic neuronal loss in this study. *In vivo* bio-distribution experiments found MPTP injection greatly changed Se bio-distribution in mice, which led to reversed alterations in the bioavailability of Se-Met and Se-Na. Se-Na had higher bioavailability than Se-Met in PD mice, which could explain its better neuroprotective effects compared to Se-Met. Our results proved that Se forms and dosages determined their biological actions in mouse models of PD. Our study will provide valuable scientific evidence to researchers and/or medical professionals in using Se for PD prevention or therapy.

## 1. Introduction

Parkinson’s disease (PD) is the second most common neurodegenerative disorder and affects about 10 million people worldwide [1]. The pathogenic mechanisms of PD are not fully clarified, but its main pathological feature is the degeneration of dopaminergic neurons in the substantia nigra pars compacta, leading to motor symptoms like rigidity, resting tremors, and postural instability in PD patients. DNA damage and oxidative stress (OS) are involved in PD pathogenesis [2]. Selenium (Se) is an essential antioxidant trace element. It is proved that Se plays a vital role in several neurodegenerative disorders, including Alzheimer’s disease and PD [3,4].

Se occurs in nature in inorganic and organic forms. Inorganic Se compounds include selenate, selenite, and selenide, as well as those naturally existing in soil and water; organic Se, such as selenomethionine (seleno-L-methionine) and selenocysteine, is synthesized in plants and animals [5]. Se performs its biological functions via synthesized selenoproteins; there are 25 selenoproteins in humans with Sec at their active center [6]. Several selenoproteins are expressed in the brain, among them glutathione peroxidases (GPxs) and selenoprotein P (Sepp1), all of which belong to important antioxidant enzymes in the brain and are highly related to PD pathology [7]. OS, an important contributor to PD pathogenesis, can particularly attack dopaminergic neurons, but most selenoproteins have antioxidant properties to protect against OS [8]. Some animal experiments have demonstrated that Se administration (in the form of inorganic Se, selenite sodium) could prevent dopaminergic cell death and decline of dopamine (DA) with its metabolites and ameliorate motor dysfunctions in PD animal models [9,10]. However, the high toxicity and unstable state of inorganic Se have limited its applications in humans. It has been found that both Se deficiency and overexposure could lead to adverse health effects or even toxicity, and different forms of Se have distinct biological properties [11].

Selenite sodium (Se-Na) and seleno-L-methionine (Se-Met) are two common Se forms used in dietary supplements for humans. Comparative effects of inorganic and organic selenium (Se) have been explored by many studies in normal animals including mice [12,13], rats [14], chickens, sheep, pigs, etc. [5]. Under normal conditions, Se-Met has higher bioavailability, with more rapid and complete (98%) absorption than Se-Na (84%) and more liver uptake in humans [15]. Concurrently, Se-Met has lower toxicity than Se-Na [16], and it is a more acceptable dietary Se [17]. Se-Na has been found to protect against the loss of dopaminergic neurons in both PD mice [18] and rat models [9,10]. Se-Met was found to play a role in preventing alpha-synuclein-induced neuron degeneration in neuroblastoma cells [19], however, no report has investigated or compared its potential neuroprotective effects with Se-Na against PD in animal models.

There is a lack of systematic studies on different Se forms used against PD and it is hypothesized that the effects of inorganic and organic Se in PD animal models might differ. In this study, the neuroprotective effects of Se-Met were systematically evaluated in both acute and chronic PD mice models in comparison to Se-Na. The mechanisms underlying their potentially differential biological actions against PD were explored.

## 2. Methods

### 2.1. Animal Experimental Design

Male C57BL/6J mice (20 ± 2 g) aged 6–8 weeks were purchased from the Laboratory Animal Services Centre of the Chinese University of Hong Kong (Hong Kong) and used in three sets of animal experiments. Animals were housed in Centralized Animal Facilities (CAF) of the Hong Kong Polytechnic University (Hong Kong). The animal experiment was approved by the Animal Subjects Ethics Sub-committee at the Hong Kong Polytechnic University (ASESC Case No.: 18-19/28-ABCT-R-HMRF). The mice were given a normal diet (AIN-93M, Harlan Laboratories Inc., Indianapolis, IN, USA) and distilled water. All the mice underwent a one-week adaptation period before entering the experiments. As reported, MPTP (1-methyl-4-phenyl-1,2,3,6-tetrahydropyridine) is a selective dopaminergic neuron toxin, the most widely used toxin to generate PD animal models [20]. Acute (one day) and chronic (5 weeks) intraperitoneal (i.p.) injections of MPTP given to mice are two classical protocols to prepare PD models. In our present study, MPTP was purchased (Sigma, St. Louis, MO, USA). According to classical protocols [20], an acute PD mice model was prepared by i.p. injections of MPTP (20 mg/kg) four times within one day, with an interval of two hours between them; a chronic PD mice model was made by i.p. injection of MPTP (20 mg/kg) twice in a week for a total of 5 weeks and the injection was performed in the mornings every Wednesday and Saturday.

#### 2.1.1. Acute PD Model Experiment

**Figure 1:**
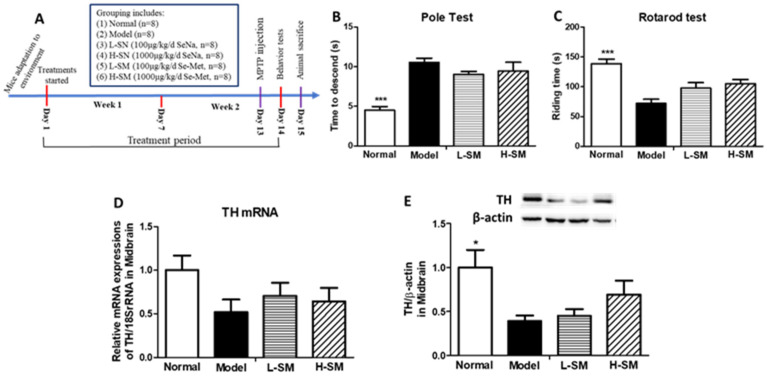
Comparative effects of Se-Na and Se-Met in acute PD mice model. (**A**) Timeline of animal experiments. (**B**) Pole test of mice indicated by total time to descent(s). (**C**) Rotarod test of mice indicated by riding time on the rod(s). TH gene (**D**) and protein (**E**) expressions in the midbrain of mice in each group. TH mRNA expressions were shown as a quantitative ratio of TH to 18S rRNA gene and relative to the group mean of the Normal group. For protein expressions, one representative band for TH and endogenous β-actin from each group; and the following bar chart shows the ratio of TH/β-actin in each group in the midbrain of mice. Values are expressed as mean ± SEM, n = 6–8. * p < 0.05, *** p < 0.001 vs. Model group. Se-Na, sodium selenite; Se-Met, Seleno-L-methionine; PD, Parkinson’s disease; TH, tyrosine hydroxylase.

After environmental adaptation, forty-eight male C57BL/6J mice (20 ± 2 g) were first used in the acute PD model experiment. Mice were randomly assigned to 6 groups with n = 8 for each group. The normal control group (Normal) and PD model group (Model) were treated with distilled water via oral gavage once daily for a total of 14 days, and on the 13th day, they were i.p. injected with saline or MPTP separately. The low dosage (L-SN), high dosage of Se-Na (H-SN), and the low (L-SM) and high (H-SM) dosage of Se-Met-treated groups were separately treated with low or high dosages of Se-Na and Se-Met via oral gavage daily for a total of 14 days, with an MPTP injection on the 13th day. The dosages of Se-Na and Se-Met were set to be 100 μg Se/kg/d (low) and 1000 μg Se/kg/d (high). Behavior tests were performed on the 14th day after the MPTP injection. On the 15th day, mice were sacrificed, and their blood was withdrawn from the heart to prepare serum. Then, both sides of the striatum and the midbrain tissues were harvested, and all samples were stored at −80 °C. The experimental timeline can be found in Figure 1A. 

#### 2.1.2. Chronic PD Model Experiment

**Figure 2:**
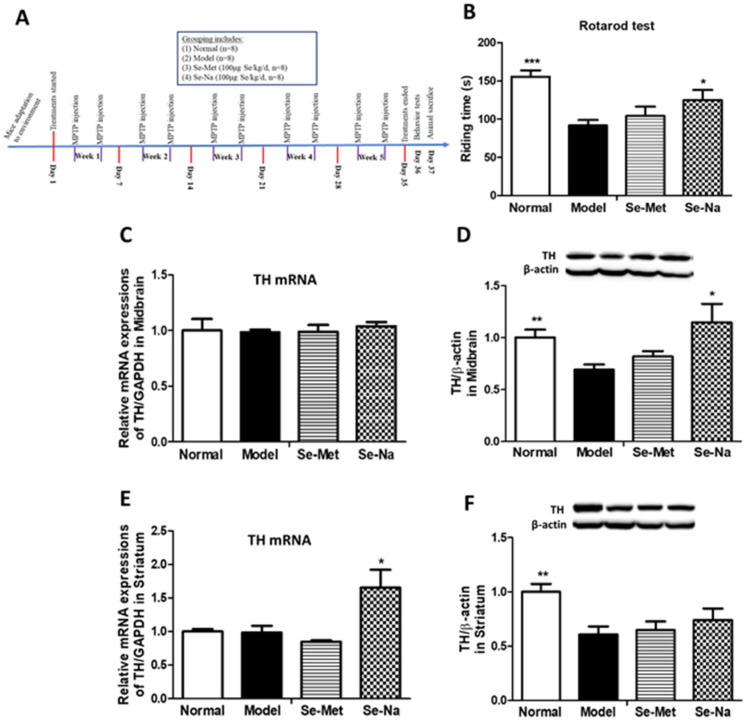
Comparative effects of Se-Na and Se-Met in chronic PD mice model. (**A**) Timeline of animal experiments. (**B**) Rotarod test of mice indicated by riding time on the rod(s). TH gene (**C**,**E**) and protein (**D**,**F**) expressions in the midbrain or striatum of mice in each group. TH mRNA expressions were shown as a quantitative ratio of TH to GAPDH gene and relative to the group mean of the Normal group. For protein expressions, one representative band for TH and endogenous β-actin from each group; and the following bar chart shows the ratio of TH/β-actin in each group in the midbrain of mice. Values are expressed as mean ± SEM, n = 8. * p < 0.05, ** p < 0.01, *** p < 0.001 vs. Model group. Se-Na, sodium selenite; Se-Met, Seleno-L-methionine; PD, Parkinson’s disease; TH, tyrosine hydroxylase.

One month after the acute PD experiment was completed, thirty-two male C57BL/6J mice (26 ± 1 g) were used in the PD chronic model experiment. The mice were randomly assigned to 4 groups with n = 8 in each group. (1) The normal control group (Normal) and (2) the PD model group (Model) were orally fed with distilled water once daily for a total of 5 weeks, together with i.p. injection of saline or MPTP twice a week; (3) The Se-Na-treated group (Se-Na), (4) the Se-Met-treated group (Se-Met) were treated with Se-Na or Se-Met together with i.p. injection of MPTP twice a week. The dosages of Se-Na and Se-Met were set at 100 μg Se/kg/d (low). On the 36th day, behavior tests were performed; and on the 37th day, the mice were sacrificed for the collection of blood to prepare serum, as well as the collection of both sides of the striatum and the midbrain tissues. All samples were stored at −80 °C. The experimental timeline can be found in Figure 2A.

#### 2.1.3. Se Bio-Distribution Experiment *In Vivo*

At the same time, in the chronic PD model experiment, one month after the acute PD experiment was completed, twelve male C57BL/6J mice (26 ± 1 g) were randomly assigned to 4 groups with n = 3 in each group. First, acute PD mice models were established, then Se-Na or Se-Met at the same Se dosage (1 mg Se/kg) was orally fed to normal or acute PD mice separately for 24 h, followed by sacrifice and collection of their blood, livers, and kidneys. The collected organs were weighed and digested in HNO_3_ and HClO_4_ (3:1) solution at a temperature of 180 °C. The homogenized tissue lysates were heated to dry, followed by dilution with ultrapure water and filtration. Se concentrations in the collected organs were determined by Inductively Coupled Plasma—Optical Emission Spectrometry (ICP-OES) after microwave digestion.

### 2.2. Behavior Tests

Pole test and rotarod test were applied in this study to appraise the motor function of the mice. The methodology to conduct the pole tests stays consistent with our previous study and is described in detail in our published paper [21]. For the rotarod test, the methods referred to others’ papers [22,23]. Briefly, after being trained twice, mice were put on the Omni Rotor (Omnitech Electronics Inc., Columbus, OH, USA), an automated accelerating rotarod apparatus with a diameter of 1.25 inches. The speed of the rod was set to accelerate from 4 rpm to 40 rpm within 2 min. The riding time on the rod for mice was recorded. The trials were repeated 3 times and the results were the average time of 3 trials.

### 2.3. Western-Blot Analysis

From the midbrain or striatum tissues, total proteins were extracted after being homogenized on ice in an ice-cold lysis buffer. Western-blot experiment methodology employed was the same as in our previously published studies [21,24]. Briefly, an equal number of proteins was separated using SDS-PAGE gels and transferred to PVDF membranes. Membranes were then incubated with non-fat milk, and primary and secondary antibodies sequentially. A chemiluminescence kit was used to visualize protein bands. Image J software was used to measure the intensity of bands. Antibodies included primary mouse anti-TH (1:1000, Millipore, Burlington, MA, USA) and mouse anti-β-actin (1:1000, Santa Cruz, Dallas, TX, USA), and a secondary antibody of goat-anti-mouse IgG (1:1000, Santa Cruz, Dallas, TX, USA). 

### 2.4. Real-Time PCR

Brain tissues (about 20 mg) were mixed with 1 mL Trizol and homogenized on ice followed by total RNA extraction. Real-time PCR was conducted in the iCycler iQ5 system (Bio-Rad Laboratories, Hercules, CA, USA) to measure the mRNA levels of target genes, and the protocols were the same as described in our previously published papers [21,25]. The expression levels of the genes for tyrosine hydroxylase (TH), GPX1, GPX4, and Selenoprotein P (Sepp1) were analyzed. Then, an 18S ribosomal RNA (18S rRNA) gene or GAPDH gene was used as the endogenous reference for normalization. Each sample was detected in duplicate. Levels of mRNA were shown as a quantitative ratio of the target gene to 18S rRNA or GAPDH gene and relative to the group mean of the Normal group. The sequences of the primers were shown in Supplementary Table S1. 

### 2.5. Microwave Digestion and ICP-OES

SRC microwave (ultraWAVE, Milestone Inc., Shelton, CT, USA) was used to perform sample digestion before Se concentration detections. Referring to protocols in the previous paper [26], samples or analytical blanks (500 μL) were added directly into the vessels and mixed with 10 mL 70% HNO_3_. The vessels were fixed on the microwave rotor, and the heating program was started: ramp for 10 min up to 180 °C and hold for 20 min at 180 °C. Then they were cooled down to room temperature and all digested samples in the vessels were transferred to a 25 mL volumetric flask and diluted to up to 25 mL with 5% HNO_3_. After microwave digestion, Se content in the samples was determined using Inductively Coupled Plasma—Optical Emission Spectrometry (ICP-OES) (Agilent Technology Inc., Santa Clara, CA, USA) with the operational conditions: radio frequency power of 1550 W, plasma gas flow rate of 3.0 L min^−1^, an auxiliary gas flow rate of 0.82 L min^−1^, nebulizer gas flow rate of 0.36 L min^−1^. The peak (*m*/*z*) Se was monitored. 

### 2.6. Statistical Analysis

Animals in the present experiment were randomly assigned to different groups and experimenters were blinded for all analysis purposes. Data were reported as mean ± SEM or mean ± SD. Statistical analyses were performed using PRISM version 9.4 (Graphpad, San Diego, CA, USA). Analyses of treatments on different parameters were conducted by one-way ANOVA followed by Tukey’s test or *t*-test as a post-test. Differences in *p* values of less than 0.05 were considered statistically significant.

## 3. Results

### 3.1. Comparative Effects of Se-Na and Se-Met in Acute PD Mice Model

During our experiments, almost all mice in L-SN and H-SN that had been treated with low and high dosages of Se-Na died after MPTP injections. This might be attributed to the higher toxicity of Se-Na compared with Se-Met. Those mice that had been treated with Se-Na for 14 days could not tolerate the final addition of MPTP neurotoxin. Moreover, animal weights in the other groups did not change much during the whole study period, showing treatment with low or high doses of Se-Met could not influence the body weight of PD mice (Table 1).

Our results showed PD model mice exhibited locomotor deficits (*p* < 0.001 vs. Normal) with longer total descent times (Figure 1B) and shorter riding times on the rod (Figure 1C). Both low and high dosages of Se-Met treatments failed to change much the descending time and riding time of PD mice in the pole and rotarod test (Figure 1B,C). Tyrosine hydroxylase (TH) expressions in the midbrain could represent the status of dopaminergic neuronal loss in PD mice. In the present study, TH gene and protein expressions were measured by real-time PCR and Western blot. The results showed that the differences in TH mRNA expressions (Figure 1D) among different groups did not reach statistical significance, TH protein expressions in PD mice declined greatly (*p* < 0.05 vs. Normal, Figure 1E), indicating that a PD mice model was successfully made. However, neither low nor high dosages of Se-Met treatment could significantly improve the TH gene or protein expressions in the midbrain of PD mice (vs. Model, Figure 1D,E).

### 3.2. Comparative Effects of Se-Na and Se-Met in Chronic PD Mice Model

The body weight of animals from each group did not change much during the whole study period, and neither Se-Met nor Se-Na could change the body weight of PD mice induced by chronic MPTP injection (Table 1). The behavior results showed that there were no obvious changes in the total descent time in the pole test among different groups (Data not shown). However, in the rotarod performance test, PD model mice made by chronic MPTP injection showed significant motor deficits as indicated by the decline in riding time on the rod (*p* < 0.01 vs. Normal, Figure 2B). Treatment with a low dosage of Se-Na, but not Se-Met, could significantly improve motor functions of chronic PD mice models by increasing their riding time on the rod (*p* < 0.05, vs. Model, Figure 2B). 

TH gene expressions in either the midbrain (Figure 2C) or the striatum (Figure 2E) in chronic PD models did not change much, but TH protein expressions declined greatly in both the midbrain (*p* < 0.01, Figure 2D) and the striatum (*p* < 0.01, Figure 2F) in PD mice (vs. Normal). This result, together with the behavior result, indicated our chronic PD mice model had been successfully prepared. Similarly, Se-Na treatment, but not Se-Met, was found to significantly increase TH protein expressions in the midbrain (Figure 2D) and increase TH gene expressions (Figure 2E) in the striatum of PD mice (*p* < 0.05, vs. Model). 

### 3.3. Comparative Effects of Se-Na and Se-Met on Gene Transcripts of Selenoproteins in the Brain

**Figure 3:**
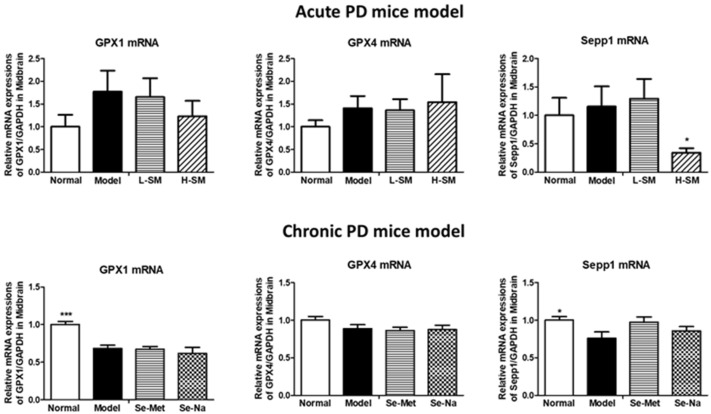
Comparative effects of Se-Na and Se-Met on gene transcripts of selenoproteins in the brain. mRNA expressions GPX1, GPX4, and Sepp1 in the midbrain of mice from both acute and chronic PD experiments. Levels of mRNA were shown as a quantitative ratio of the target gene to the GAPDH gene and relative to the group mean of the Normal group. Values are expressed as mean ± SEM, n = 6–8. * p < 0.05, *** *p* < 0.001 vs. Model group. Se-Na, sodium selenite; Se-Met, Seleno-L-methionine; GPX1, glutathione peroxidase 1; GPX4, glutathione peroxidase 4; Sepp1, selenoprotein P; PD, Parkinson’s disease.

Se plays its role mainly through synthesized selenoproteins in the organisms. In this study, we detected mRNA levels of three major selenoproteins (GPX1, GPX4, and Sepp1) that were involved in PD in the midbrain by real-time PCR. As shown in Figure 3, all the detected selenoprotein mRNA levels seemed to have increased in response to acute injections of MPTP in the acute PD mice model, but it did not reach statistical significance. However, in response to chronic MPTP injections, PD mice had lower mRNA levels of selenoproteins, GPX1 (*p* < 0.001), and Sepp1 (*p* < 0.05) in particular (vs. Normal). In acute PD mice, Se-Met at either low or high doses could not influence much the mRNA levels of GPX1 and GPX4, but treatment with a high dosage of Se-Met significantly downregulated Sepp1 mRNA levels (*p* < 0.05 vs. Model). There was no obvious influence seen with the same Se dose of Se-Na and Se-Met on the mRNA levels of these three selenoproteins in the chronic PD model (Figure 3). 

### 3.4. Comparative Effects of Se-Na and Se-Met on GPX Activities in the Brain

**Figure 4:**
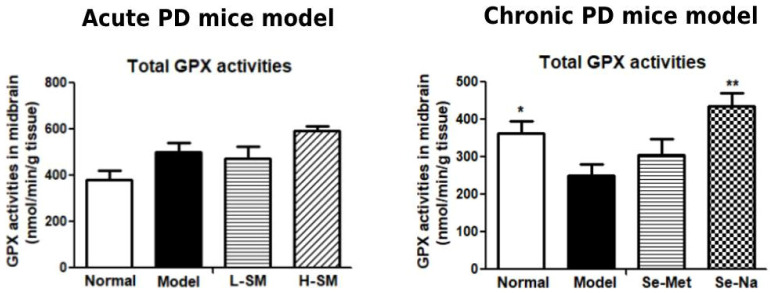
Comparative effects of Se-Na and Se-Met on GPX activities in the brain. Total GPX activities in the midbrain of mice from both acute and chronic PD experiments were measured by commercial ELISA kit and expressed as nmol/min/g tissue. Values are expressed as mean ± SEM, n = 6–8. * *p* < 0.05, ** *p* < 0.01 vs. Model group. Se-Na, sodium selenite; Se-Met, Seleno-L-methionine; GPX, glutathione peroxidase; PD, Parkinson’s disease.

As the gene expression levels of major selenoproteins did not change much in response to Se-Na or Se-Met, we measured total GPX activities in the midbrain of mice by using a commercial ELISA kit. The results are shown in Figure 4. It was found that total GPX activities appeared to increase in acute PD mice, but significantly declined in chronic PD mice (*p* < 0.05 vs. Model). Both low and high-dosage Se-Met treatments in acute PD mice failed to induce total GPX activities, but Se-Na at low dosage was found to greatly improve GPX activities in chronic PD mice (*p* < 0.01 vs. Model).

### 3.5. Bio-Distribution of Se-Na and Se-Met in Normal and Acute PD Model Mice

We orally fed the same Se dose (1 mg Se/kg) of Se-Na and Se-Met to normal and acute PD mice simultaneously for 24 h. The bio-distribution of Se was quantified in the serum, livers, and kidneys of mice by ICP-OES and the results were summarized in Table 2. It was found that Se contents in the serum and liver of normal mice treated with Se-Met were higher than those in normal mice treated with Se-Na (*p* < 0.01), indicating Se-Met had higher bioavailability than Se-Na under normal conditions. However, in PD mice, serum Se levels were higher (*p* < 0.001), and liver Se levels (*p* < 0.01) were lower in response to Se-Na than Se-Met treatments, suggesting that Se-Na might have higher bioavailability than Se-Met in PD mice. From another point of view, Se bio-distribution in response to either Se-Met or Se-Na supplementation obviously differed in normal and PD mice. Serum Se levels were significantly lower (*p* < 0.01) in Se-Met-treated PD mice than in Se-Met-treated normal mice; but conversely, serum Se levels were much higher (*p* < 0.001) in Se-Na-treated PD mice than its treated normal mice (Normal vs. PD mice). A similar Se content in the liver of normal and PD mice was found in either Se-Met- or Se-Na-treated groups (Normal vs. PD mice). However, both Se-Met (*p* < 0.001) and Se-Na (*p* < 0.05) oral treatments resulted in lower Se levels in the kidney of PD mice compared with those in normal mice (Normal vs. PD mice).

## 4. Discussion

Inorganic Se has been reported to exert neuroprotective effects against neurotoxins (MPTP or 6-hydroxydopamine) induced neuron injuries in PD animal models [9,10,18], however, many researchers found the range is so narrow between its effective and toxic dose, and its effects against PD are not consistent in different labs. Moreover, there are not yet any reports on the comparative effects of organic Se with inorganic Se against PD. Our present study filled in this gap to investigate the possible neuroprotective effects and underlying mechanism of organic Se (Se-Met) in comparison with inorganic Se (Se-Na) in two classical PD mice models.

Our results found the high toxicity of inorganic Se-Na had led to the deaths of most of the animals even at a relatively lower dosage (100 μg Se/kg/d) treatment for two weeks when it was administered together with the acute injection of MPTP neurotoxin. However, organic Se-Met showed lower toxicity with fewer animal deaths even at higher dosages (1000 μg Se/kg/d for two weeks) in the acute PD mice models. This result confirmed again the fact that Se-Met had lower toxicity than Se-Na, which is why Se-Met is widely used in dietary supplements [16,17]. However, our results showed that the oral administration of Se-Met at both low and high dosages for two weeks failed to prevent dopaminergic neuronal loss in the acute PD mice, as evidenced by the behavior results and TH expressions in mice midbrain. Se-Met was ever reported to prevent alpha-synuclein-induced neuron degeneration in neuroblastoma cells [19], however, there are not yet any reports that investigate and compare its potential neuroprotective effects with Se-Na in PD animal models. Hereby, we further investigated the potential neuroprotective effects of Se-Met in chronic PD mice models in comparison with Se-Na. Our results showed long-term oral administration of Se-Met at low dosage failed to improve motor functions and prevent dopaminergic neuronal loss in PD mice. However, consistent with previous publications, oral administration of a low dosage of Se-Na over five weeks exerted significant neuroprotective effects against MPTP-induced dopaminergic neuronal loss, as evidenced by improvement of motor functions and TH expressions in the midbrain and striatum of PD mice. These results strongly suggested that Se forms and dosages determined their actions against PD. 

Se exerts its biological functions in humans mainly via 25 selenoproteins [6], among which GPxs and Sepp1 are important antioxidant enzymes in the brain and highly related to PD pathology [7]. Previous studies have reported that alterations to selenoproteins mRNA levels in response to Se deficiency are tissue-specific in animals [27,28]. Zhang et al. measured the mRNA levels of in total, 24 types of selenoproteins in five brain regions including substantia nigra using real-time PCR in a chronic PD mice model, and their results demonstrated a wide range of changes of selenotranscriptome in PD mice in a manner depending on selenoprotein type and brain regions [29]. In particular, GPX1, GPX4, and Sepp1 are abundant in five brain regions, and they have been found to decline significantly in the substantia nigra of PD mice compared to normal ones [29]. Consistently, our results showed that mRNA expressions of these selenoproteins and total GPX activities declined greatly in chronic PD mice, but no significant changes were found in acute PD mice. 

Se deficiency may increase while excess Se intake may inhibit selenoprotein expressions or their activities [30]. Excess Se intake can impair the antioxidant defense mechanisms by generating oxygen-free radicals [31]. Our results showed that organic Se-Met supplementation did not much influence GPX mRNA levels and activities in the midbrain of either acute or chronic PD mice, and high dose of Se-Met treatment even led to downregulation of Sepp1 mRNA levels in the midbrain of acute PD mice. These results suggested that the high dose of Se-Met used in acute PD mice was already excessive. In comparison, oral supplementation of inorganic Se-Na at a low dose did not significantly affect mRNA levels of three selenoproteins but it significantly improved total GPX activities in the midbrain of chronic PD mice. It has been reported that the levels of selenoproteins mRNA may not be in parallel with the changes in their activities in response to Se deficiency or supplementation, and a post-transcript control might be involved to keep mRNA stable in this process [32]. This can explain our present result that Se-Na treatment could influence GPX activities but not their mRNA expressions. Enzyme activities are more important than their expressions in organisms to play antioxidant roles. The enhanced GPX activities by Se-Na, but not Se-Met, in PD mice could be the major reason for the positive actions of inorganic Se in the prevention of dopaminergic neuronal loss in this study. 

Se-Met has higher bioavailability under normal conditions, with more rapid and complete (98%) absorption than Se-Na (84%) and more liver uptake in humans [15]. However, our results clearly demonstrated that Se-Na provided better neuroprotective effects in MPTP-induced PD mice than Se-Met at the same Se dosage. Hence, an *in vivo* biodistribution experiment of Se-Met and Se-Na (at the same Se dosage) was employed in our study to compare their potential differences in oral bioavailability in both normal and MPTP-induced acute PD mice. Our results showed that the oral administration of Se-Met had higher distribution than Se-Na to blood and liver of normal mice, suggesting higher bioavailability of Se-Met under normal conditions, a result that was consistent with previous findings [15,33]. However, when Se-Met or Se-Na was administered to PD mice induced by MPTP injection, Se bio-distribution changed greatly within 24 h. In PD mice, serum Se levels declined significantly after supplementation of Se-Met, but increased greatly after Se-Na supplementation, a converse result with those in normal mice. This indicated that Se-Na had higher bioavailability than Se-Met in PD mice. Furthermore, Se distribution in the liver did not change much, but its distribution to the kidney declined after Se-Met or Se-Na supplementation in PD mice (vs. normal mice). Herein, in either normal or PD mice, Se-Met treatment had higher liver Se distribution than Se-Na but similar kidney Se distribution. This result indicated that MPTP injection influenced Se bio-distribution likely via regulating Se absorption and excretion, but that it had no influence on its storage in the liver; such changes in PD mice altered the metabolism of different Se forms leading to reversed alterations of bioavailability between Se-Met and Se-Na. Until now, there has been no report on Se bioavailability status in PD mice. Our novel finding in the present study can explain, at least in part, why Se-Na had better neuroprotective effects than Se-Met in PD mice.

Our study is limited to observing the comparative effects of inorganic and organic Se in two MPTP-induced PD mice models and exploring the possible mechanisms for their differential actions. Our findings were novel and helpful to fill in a knowledge gap. However, comparisons with the other PD models, even in humans, might be needed in the future to confirm our present results. Additionally, the bio-distribution experiment was only performed in acute PD models at a single time point in our present work. Future work is needed to systematically compare the absorption and metabolism process for inorganic and organic Se across different PD animal models at multiple time points to provide more solid evidence for use of Se in the management of PD. 

## 5. Conclusions

Se-Na provided better neuroprotective effects in PD mice than Se-Met at the same but relatively low Se dosage. Se-Na treatment could influence GPX activities but not their mRNA expressions in the midbrain of PD mice. The enhanced GPX activities by Se-Na, but not Se-Met, in PD mice could be the major reason for the positive actions of inorganic Se to prevent dopaminergic neuronal loss in this study. *In vivo* bio-distribution experiment found MPTP injection greatly changed the Se bio-distribution in mice, leading to reversed alterations in the bioavailability of Se-Met and Se-Na. Se-Na had higher bioavailability than Se-Met in PD mice, which could explain its better neuroprotective effects than Se-Met. Our results proved the forms and dosages of Se were determining factors for their biological actions in mouse models of PD. Our study will provide valuable scientific evidence to researchers and/or medical professionals in the use of Se for the management of PD. Based on our present results, inorganic Se at a low dose might be more appropriate to be applied in the prevention and/or therapy of PD.

## Figures and Tables

**Table 1:** Body weight and final animal numbers in each group.

			Body Weight	Body Weight
	Groups	N	Beginning (g)	End (g)
Acute PD mice model	Normal	8	19.3 ± 0.5	22.5 ± 0.3
	Model	6	19.8 ± 0.5	22.6 ± 0.4
	L-SM	7	20.1 ± 0.6	22.3 ± 0.4
	H-SM	6	19.5 ± 0.6	22.2 ± 0.6
Chronic PD mice model	Normal	8	27.1 ± 0.5	26.9 ± 0.6
	Model	8	26.4 ± 0.3	25.8 ± 0.4
	Se-Met	8	26.2 ± 0.9	25.8 ± 0.9
	Se-Na	8	26.8 ± 0.7	26.0 ± 0.6

Values are expressed as mean ± SEM. N represents the numbers in each group. PD, Parkinson’s disease. Grouping in acute PD mice model includes: Normal, Model, low (L-SM) and high (H-SM) dosage of seleno-L-methionine treated groups. Grouping in chronic PD mice model includes: Normal, Model, low dosage of seleno-L-methionine (Se-Met) and sodium selenite (Se-Na) treated groups.

**Table 2:** Comparison of Se bio-distribution in normal and PD mice after supplementation of different Se forms.

	Se Concentration in Different Tissues	Se-Met (1 mg Se/kg)	Se-Na (1 mg Se/kg)
Normal mice	Serum (µg/mL)	1.55 ± 0.32	0.31 ± 0.11 **
	Liver (µg/g)	3.16 ± 0.44	1.72 ± 0.25 **
	Kidney (µg/g)	2.70 ± 0.17	3.03 ± 0.52
Acute PD model mice	Serum (µg/mL)	0.59 ± 0.07 ^##^	5.59 ± 0.84 ***^###^
	Liver (µg/g)	2.77 ± 0.19	1.90 ± 0.13 **
	Kidney (µg/g)	1.75 ± 0.02 ^###^	1.52 ± 0.41 ^#^

Values are expressed as mean ± SD, n = 3. ** *p* < 0.01, *** *p* < 0.001 vs. Se-Met treated normal or acute PD mice; ^#^ *p* < 0.05, ^##^ *p* < 0.01, ^###^ *p* < 0.001 vs. normal mice treated by Se-Met or Se-Na. PD, Parkinson’s disease; Se, selenium; Se-Na, sodium selenite; Se-Met, seleno-L-methionine. Se-Met and Se-Na were fed to normal or acute PD model mice via oral gavage at the same Se dose (1 mg Se/kg) for 24 h followed by collection of different tissues for Se concentration measurement.

## Data Availability

Data will be made available upon request.

## Supplementary Materials

The following supporting information can be downloaded at: https://www.mdpi.com/article/10.3390/nu15010011/s1, Figure S1: Original images of western blot (WB) bands; Table S1: Primer list for real time RT-PCR analysis. Reference [34] is cited in the Supplementary Materials.
