# Supplementary material for: Selenium Forms and Dosages Determined Their Biological Actions in Mouse Models of Parkinson’s Disease
Source: Nutrients. 2022 Dec 20;15(1):11. doi: 10.3390/nu15010011 (PMC9824164; doi:10.3390/nu15010011)
Supplement: Supplementary file 1 [file nutrients-15-00011-s001.zip › nutrients-2072101-supplementary.pdf]

Supplementary Materials

Selenium Forms and Dosages Determined Their Biological Actions in Mouse Models of Parkinson's Disease

Chongchong Sun [1], Zhongrui Du [2,3,4,5], Xin Liu [2,3,4], Ye Yang [3,4], Sainan Zhou [2,3,4], Chong Li [3,4], Xu Cao [6], Qing Zhao [7], Kahing Wong [2,3,4], Wenfang Chen [1,*] and Xiaoli Dong [2,3,4,*]

[1] Department of Physiology, School of Basic Medicine, Qingdao University, Qingdao 266071, China
[2] The Hong Kong Polytechnic University Shenzhen Research Institute, Shenzhen 518057, China
[3] Research Institute for Future Food, The Hong Kong Polytechnic University, Hong Kong 852, China
[4] Department of Applied Biology and Chemical Technology, The Hong Kong Polytechnic University, Hong Kong 852, China
[5] BioNanotechnology Institute, Ludong University, Yantai 264025, China
[6] Department of Neurology, Shenzhen University General Hospital, Shenzhen University Clinical Medical Academy, Shenzhen 518055, China
[7] Department of Neurology, Linzi Maternal & Child Health Hospital of Zibo, Zibo 255400, China
* Correspondence: chenwenfang@qdu.edu.cn (W.C.); xiaoli.dong@polyu.edu.hk (X.D.); Tel.: +86-532-85956866 (W.C.); +852-34008668 (X.D.); Fax: 852-23649932 (X.D.)

Table S1. Primer list for real time RT-PCR analysis.

Gene	**Forward Primer (5′-3′)**	**Reverse Primer (5′-3′)**
**TH** [1]	CCCAAGGGCTTCAGAAGAG	GGGCATCCTCGATGAGACT
**GPX1** [2]	TGGACTGGTGGTGCTCG	CGTCACTGGGTGTTGGC
**GPX4** [2]	ACGATGCCCACCCACT	CACGCAGCCGTTCTT
**Sepp1** [2]	GGCCGTCTTGTGTATCACCT	TGGTGTTTGTGGTGGCTATG
**18S rRNA** [1]	TCGGAACTGAGGCCATGATT	TTTCGCTCTGGTCCGTCTTG
**GAPDH** [3]	CATGGCCTTCCGTGTTCCTA	CCTGCTTCACCACCTTCTTGAT

Note: the primer sequences for these genes refer to previously published papers as shown in this Table. Abbreviations: TH, tyrosine hydroxylase; GPX1, glutathione peroxidase 1; GPX4, glutathione peroxidase 4; Sepp1, selenoprotein P

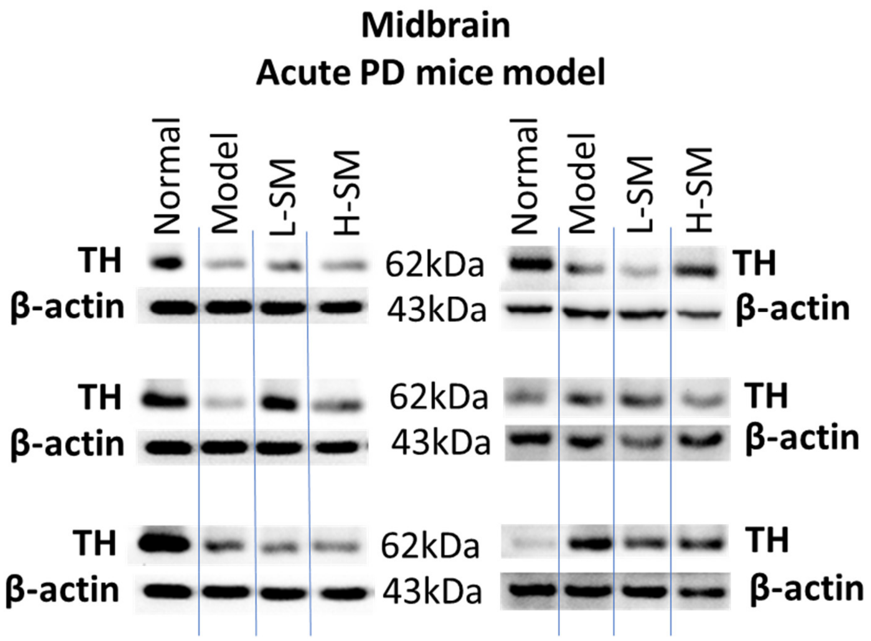
Midbrain
Acute PD mice model
Normal
Model
L-SM
H-SM
TH
β-actin
62kDa
43kDa

(**A**)

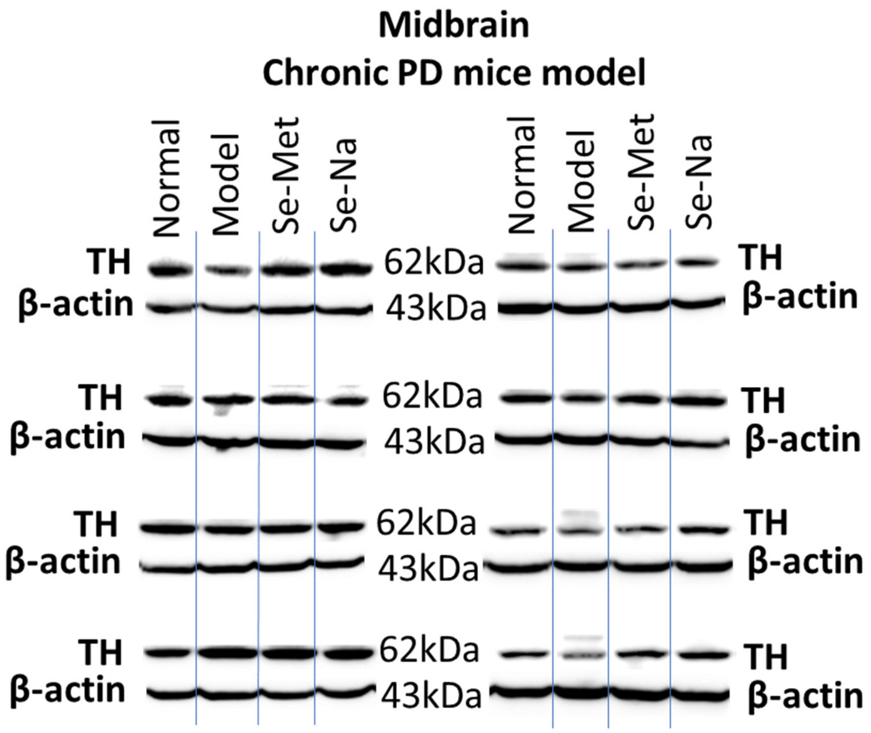
Midbrain
Chronic PD mice model
Normal
Model
Se-Met
Se-Na
TH
β-actin
62kDa
43kDa

(**B**)

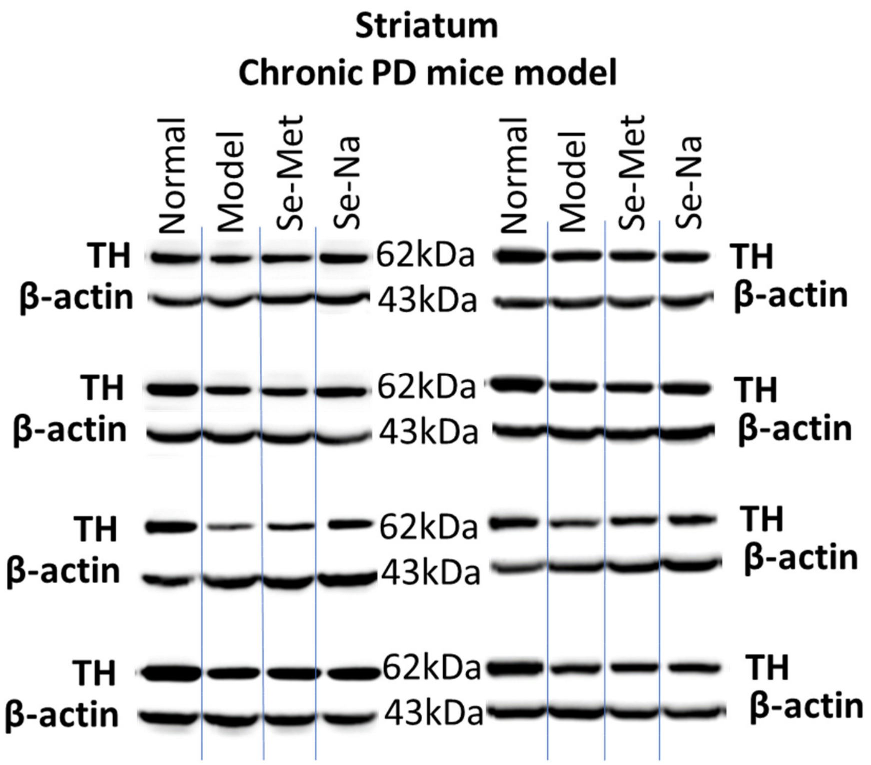

(**C**)

Figure S1. Original images of western blot (WB) bands to display protein expressions of tyrosine hydroxylase (TH) and its endogenous control (β-actin) in the midbrain of each mouse from acute PD model experiment (A); and the midbrain (B) or striatum (C) of each mouse from chronic PD model experiment. Grouping in acute PD mice model includes: Normal, Model, low (L-SM) and high (H-SM) dosage of seleno-L-methionine treated groups. Grouping in chronic PD mice model includes: Normal, Model, low dosage of seleno-L-methionine (Se-Met) and sodium selenite (Se-Na) treated groups.

References

1. Liu, X.; Du, Z.R.; Wang, X.; Sun, X.R.; Zhao, Q.; Zhao, F.; Wong, W.T.; Wong, K.H.; Dong, X.L. Polymannuronic acid prebiotic plus Lacticaseibacillus rhamnosus GG probiotic as a novel synbiotic promoted their separate neuroprotection against Parkinson's disease. *Food Res. Int.* **2022**, *155*, 111067.
2. Zhang, X.; Ye, Y.L.; Zhu, H.; Sun, S.N.; Zheng, J.; Fan, H.H.; Wu, H.M.; Chen, S.F.; Cheng, W.H.; Zhu, J.H. Selenotranscriptomic Analyses Identify Signature Selenoproteins in Brain Regions in a Mouse Model of Parkinson's Disease. *PLoS ONE* **2016**, *11*, e0163372.
3. Yu, S, Luk, KH, Cheung, ST, Kwok, KW, Wong, KH, Chen, T: Polysaccharide-protein complex-decorated selenium nanosystem as an efficient bone-formation therapeutic. *J. Mater. Chem. B* **2018**, *6*, 5215–5219.
